# Neuromyocysticercosis Causing Lateral Rectus Palsy

**DOI:** 10.4269/ajtmh.15-0068

**Published:** 2015-10-07

**Authors:** Venugopalan Y. Vishnu, Chirag K. Ahuja, Vivek Lal

**Affiliations:** Department of Neurology, Post Graduate Institute of Medical Education and Research (PGIMER), Chandigarh, India; Department of Radiology, Post Graduate Institute of Medical Education and Research (PGIMER), Chandigarh, India

**Figure 1.**
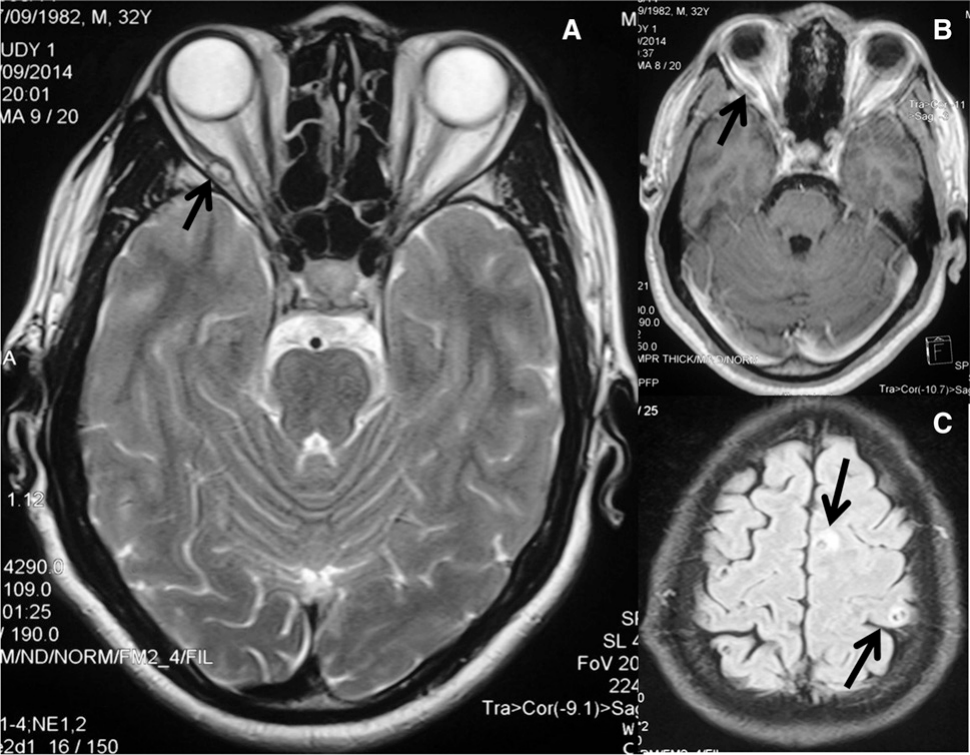
(**A**) T2W cranial magnetic resonance imaging (MRI) showing cystic lesion in the right lateral rectus with an eccentric scolex. (**B**) Contrast-enhanced cranial MRI showing minimal patchy enhancement of the lesion the right lateral rectus. (**C**) T2W cranial MRI showing multiple cysts with scolices and perilesional edema at high frontal brain parenchyma.

A 32-year-old nonvegetarian man from a known cysticercosis-endemic region of north India presented with blurriness on looking to the right. Covering one eye restored normal vision. Visual acuity and color vision were normal. He had had two generalized seizures in the previous 2 years for which he had not sought treatment. On examination, he could not abduct the right eye, considered to be a right lateral rectus palsy but no other neurological deficit was apparent. Fundus examination was normal. Cranial magnetic resonance imaging (MRI) showed two well-defined lesions in the brain parenchyma representing degenerating cysts with surrounding inflammation. A cystic lesion was visualized in the right lateral rectus muscle. A diagnosis of neuromyocysticercosis was established (Figure 1A–C

). Serum immunoglobulin (IgG) cysticercal antibodies (enzyme-linked immunosorbent assay) were positive (> 1/800). He was treated with tapering prednisone and albendazole (400 mg bid × 14 days).^1^ Carbamazepine, initiated for seizures, and prednisone were tapered off in 1 month.^1^ Diplopia disappeared 3 months after treatment was initiated, and there was no further seizure. This case depicts that extraocular muscle abnormality can manifest as visual alteration in patients with a history of seizures in cysticercosis-endemic regions.^1^
